# Role of Herbal Extracts of Catechu from *Uncaria gambir* in the Treatment of Chronic Diabetic Wounds

**DOI:** 10.3390/ph16010066

**Published:** 2022-12-31

**Authors:** Tsung-Jung Ho, Pei-Hsuan Tsai, Chia-Ho Hsieh, Jung-Hsing Lin, Yu-Wei Lin, Jia-Ru Wu, Hao-Ping Chen

**Affiliations:** 1 Integration Center of Traditional Chinese and Modern Medicine, Hualien Tzu Chi Hospital, Hualien 97002, Taiwan; 2 Department of Chinese Medicine, Hualien Tzu Chi Hospital, Hualien 97002, Taiwan; 3 School of Post-Baccalaureate Chinese Medicine, Tzu Chi University, Hualien 97004, Taiwan; 4 Department of Biochemistry, School of Medicine, Tzu Chi University, Hualien 97004, Taiwan

**Keywords:** angiogenesis, catechu, herbal medicine, Jinchuang ointment, wound healing

## Abstract

Catechu is a dried decoction from twigs with the leaves of *Uncaria gambir*. Its antioxidant, anti-inflammatory, and antimicrobial activities have been previously reported because of its high catechin and epicatechin content (>21%). It is also one of the components used in traditional Chinese herbal medicine, “Jinchuang Ointment,” which has excellent efficacy in treating chronic diabetic wounds. An in vivo zebrafish embryo platform and an in vitro cell-based tube formation assay were used to measure the angiogenic activity of catechu extracts. Interestingly, for the first time, catechu extracts stimulated angiogenic activity on both platforms. The expression of the IL-8 gene was induced in HMEC1 cells after treatment with catechu extracts for 1 h only. In contrast, the upregulation of FGFR2, FGFR3, NF-κB, STAT3, and vimentin persisted for 24 h. A summary of the possible mechanisms underlying the angiogenic activity of catechu extracts in HMEC1 cells is shown. Unexpectedly, catechu extracts inhibited the migration of HaCaT cells. These results can account for the intense blood flow flux in porcine excisional wound sites in our previous studies, which provides insights into the therapeutic activity of catechu extract in chronic diabetic wounds.

## 1. Introduction

The traditional Chinese herbal medicine, “Jinchuang ointment,” has excellent efficacy in the treatment of chronic non-healing wounds in patients with diabetes [1]. Its efficacy was also examined and confirmed in a porcine excisional wound model [2]. This herbal medicine is also effective for ulcers in people with leprosy (Hansen’s disease) [3]. This ointment contains lard, wax, starch, borneol, camphor, frankincense, dragon blood, myrrh, and catechu. Dragon blood is a red resin from the palm tree *Daemonorops draco*, grown in southeast Asia [4]. It possesses intense angiogenic activity, as confirmed by a cell-based tube formation assay and an in vivo zebrafish embryo assay [5,6]. The primary active molecule responsible for the angiogenic activity in this red resin is dracorhodin. The content of dracorhodin in dragon blood should generally be higher than 1%, as stated in Pharmacopeia [7,8].

**Figure 1:**
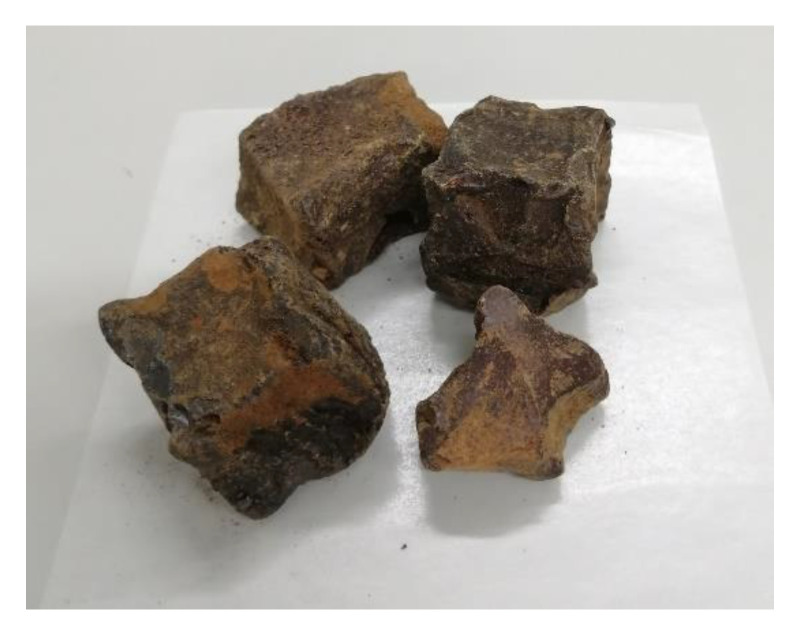
Appearance of catechu blocks.

Catechu is a dried decoction from twigs with leaves of *Uncaria gambir* Roxb. (Family Rubiaceae) or the peeled branches and stems of *Acacia catechu* (L.f.) Willd, (Family Leguminosae) [7]. The origins of *Uncaria gambir* Roxb. are Myanmar, Indonesia, and India, and that of *Acacia catechu* (L.f.) Willd is the province of Yunnan and Guanxi in China [9]. As stated in the Taiwan Herbal Pharmacopeia and Pharmacopeia of the People’s Republic of China, the total amount of catechin and epicatechin in catechu should be >21.0% [7,10]. However, the Pharmacopeia of the People’s Republic of China reported that catechu was prepared from *Acacia catechu* only. Previous studies have reported catechu from *Uncaria gambir* Roxb. It possesses antioxidant [11], anti-inflammatory [12], and antimicrobial activities [13]. Unsurprisingly, the aforementioned biological activities of catechu mainly result from catechin and epicatechin [14]. It appears as irregular blocks with a dark brown color. Figure 1 shows that each catechu block’s composition is not homogeneous. The content of bioactive molecules may vary among each block, even for the same batch sample.

Our previous results indicated that the Jinchuang ointment-treated group had a much stronger blood flow flux in porcine excisional wound sites than the control group, as shown by laser Doppler imaging analysis [2]. First, we attributed these results to the angiogenic activity of dragon blood during wound healing. However, the content of dracorhodin in dragon blood used in that experiment was only 0.06%, much lower than the 1% content required in the Pharmacopeia! Therefore, this result implies that other components of the Jinchuang ointment might also possess angiogenic activity. Here, for the first time, we report the biological activities of catechu from *Acacia catechu* in angiogenesis, cell proliferation, and migration. These results provide a scientific basis for the efficacy of Jinchuang ointment in the treatment of non-healing wounds.

## 2. Results and Discussions

### 2.1. Determination of Catechin and Epicatechin Content in Catechu Samples from Uncaria gambir

The catechin and epicatechin contents in catechu samples were determined using HPLC analysis. Calibration standard curves of catechin and epicatechin were constructed over the concentrations investigated, from 500 μg/mL to 10 μg/mL for catechin and 500 μg/mL to 25 μg/mL for epicatechin. The correlation coefficients (R^2^) of catechin and epicatechin were 0.9986 and 0.9968, respectively. The mass percentages of catechin and epicatechin in catechu samples were 28.5% and 1.4%, respectively. Therefore, the contents of both compounds in this sample were higher than 21.0%, which is consistent with Pharmacopeia standards.

### 2.2. Catechu Extract-Induced Pro-Angiogenic Effects in Zebrafish Embryos

**Figure 2:**
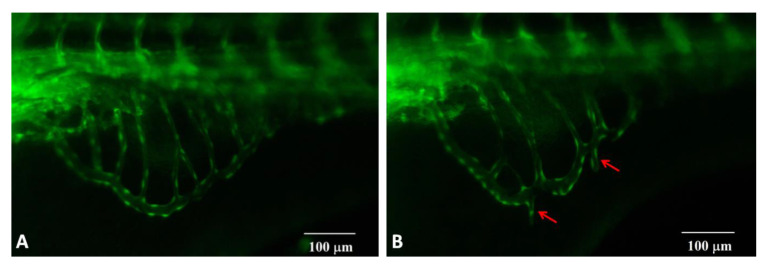
The pro-angiogenic effects on zebrafish embryos in the presence of 300 μg/mL catechu extracts. (**A**) Control, and (**B**) Catechu extract-treated. Red arrows indicate the formation of extra sprouts in the sub-intestinal veins of fish embryos. Sprout blood vessel number and blood vessel length are listed. About twenty embryos were used for each group. The measured blood vessel length unit (px) is defined using the software WenXin WximageView.

The possible toxic effects of crude catechu extracts on zebrafish embryos were first examined by treating embryos with catechu extracts at concentrations ranging from 150 μg/mL to 1250 μg/mL. As shown in Table 1, none of the embryos died in the presence of 300 μg/mL catechu extract. Therefore, zebrafish embryos were treated with 300 μg/mL catechu extract from 55 to 72 hpf. The development of the sub-intestinal vein (SIV) was examined and photographed at 72 hpf. As shown in Figure 2, the formation of extra sprouts in the sub-intestinal veins of fish embryos could be observed under a fluorescent microscope during their development stage (Figure 2B, red arrowheads). The number of newly formed sprout vessels and their corresponding length were measured in each embryo (Table 2). Apparently, crude catechu extracts can stimulate new blood vessel formation. Due to the toxic effect of crude catechu extracts, as shown in Table 1, the concentration of 300 μg/mL catechu extract was the maximum concentration used to treat fish embryos in this study. Neither catechin nor epicatechin possessed angiogenic activity in zebrafish embryos, even though their content was as high as 29% (data not shown).

**Table 1:** The toxicity of crude catechu extracts on zebrafish embryos. Zebrafish embryos were treated with various concentrations of crude catechu extracts, from 1250 μg/mL to 150 μg/mL. About twenty embryos were used for each experiment.

Catechu Conc. (μg/mL)	1250	1000	800	600	500	400	300	150	Control
Embryo survival rate	0%	0%	0%	0%	40%	60%	100%	100%	100%

**Table 2:** The pro-angiogenic effects on zebrafish embryos in the presence of 300 μg/mL catechu extracts. Blood vessel number and blood vessel length are listed. About twenty embryos were used for each group. The measured blood vessel length unit (px) is defined using the software Image J.

Blood Vessel Number	Control	Catechu Extract-Treated
0	13 (65%)	8 (34.8%)
1	7 (35%)	10(43.5%)
2	0 (0.0%)	5 (21.7%)
3	0 (0.0%)	0 (0.0%)
**Blood Vessel Length (px)**	**Control**	**Catechu Extract-Treated**
0	13 (65%)	8 (34.8%)
1–200	5 (25%)	7 (30.4%)
201–500	2 (10%)	8 (34.8%)
>500	0 (0.0%)	1 (6.7%)

### 2.3. In Vitro Tube Formation Angiogenic Assay

**Figure 3:**
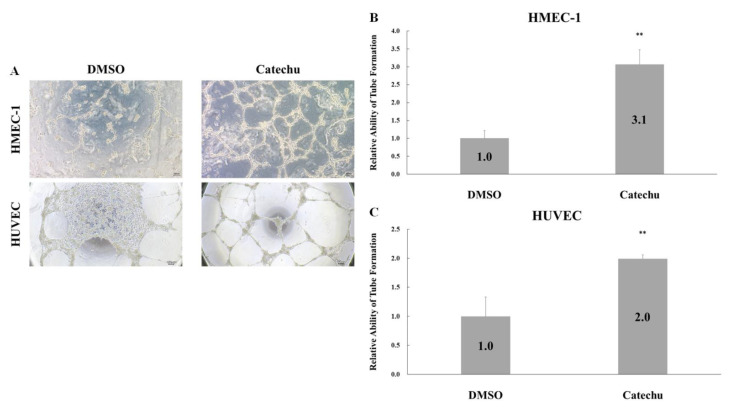
Effect of catechu extracts on the tube and network formation of HMEC-1 and HUVEC cells. (**A**) Representative photos of the tube formation assay at 24 h. HMEC-1 and HUVEC cells were treated with 0.8 ng/mL and 1.0 ng/mL crude catechu extracts, respectively. The network formation was quantified by the mesh number formation for (**B**) HMEC-1 and (**C**) HUVEC cells. Data are presented as means ± SD in three independent experiments. The *p*-value was calculated versus the control using one-tailed test analysis; *p* < 0.005 is denoted as **.

There are three stages in the wound healing process: inflammatory, proliferative, and remodeling. New blood vessel formation is essential in the proliferative stage because it is responsible for oxygen and nutrient supply, and metabolic and tissue repair waste disposal [15]. The angiogenic activity of the crude catechu extracts was also examined using an in vitro tube formation assay. HUVECs and HMEC-1 cells were used in the present study. As shown in Figure 3, the mesh number of the catechu-treated group was significantly higher than that of the control group in both cell types (** *p* < 0.005). Catechu extracts promoted two different endothelial cell capillary tubes and network formation.

### 2.4. Reverse Transcription and Quantitative Polymerase Chain Reaction

**Figure 4:**
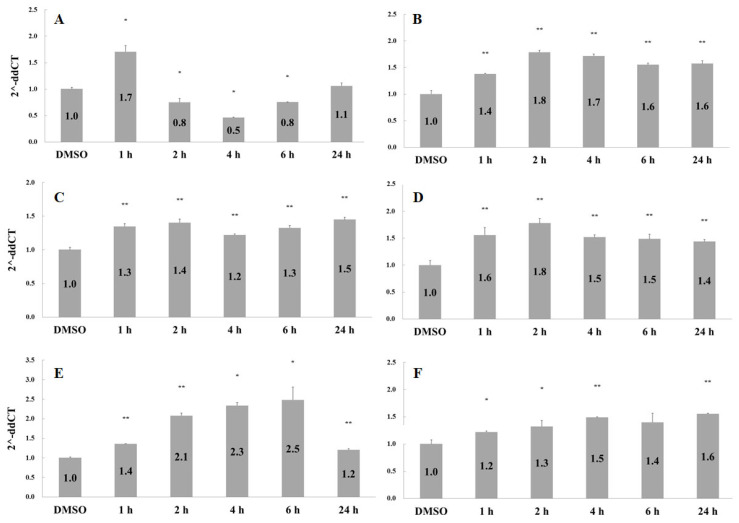
Effects of catechu extracts on gene expression. HMEC-1 cells were treated with 0.1 ng/mL of catechu extracts for 1, 2, 4, 6, and 24 h. The gene expression levels for (**A**) IL-8, (**B**) FGFR2, (**C**) FGFR3, (**D**) NF-kB, (**E**) STAT3, and (**F**) vimentin were examined by quantitative PCR. Data are presented as means ± SD in three independent experiments. The *p*-value was calculated versus the control. Using one-tailed test analysis, * indicates *p* < 0.05 and ** indicates *p* < 0.005 between different times after catechu treatment. The DMSO group was the solvent control.

The possible molecular mechanisms underlying catechu-induced angiogenesis were also investigated. IL-8 is a potent chemoattractant for neutrophils involved in the antigenic response. Meanwhile, the IL-8 protein has been found to act as an inducer for the proliferation of endothelial cells [16,17,18]. Similar to autocrine growth and angiogenic factors, IL-8 can induce epithelial-mesenchymal transition (EMT) in various cells [19,20,21]. As shown in Figure 4A, catechu treatment induces IL-8 expression at 1 h.

In contrast, upregulation of FGFR2, FGFR3, NF-kB, STAT3, and vimentin genes by catechu treatment persisted for 24 h (Figure 4B–F). The binding of fibroblast growth factor (FGF) to the FGF receptor (FGFR) on endothelial cells can trigger several cellular processes such as proliferation, gene expression of growth factors, and angiogenesis [22,23,24,25]. Moreover, STAT3 signaling can be activated by FGFRs [26]. Both NF-kB and STAT3 are transcription factors involved in various signaling pathways that lead to cellular growth, proliferation, motility, adhesion, and angiogenesis.

**Figure 5:**
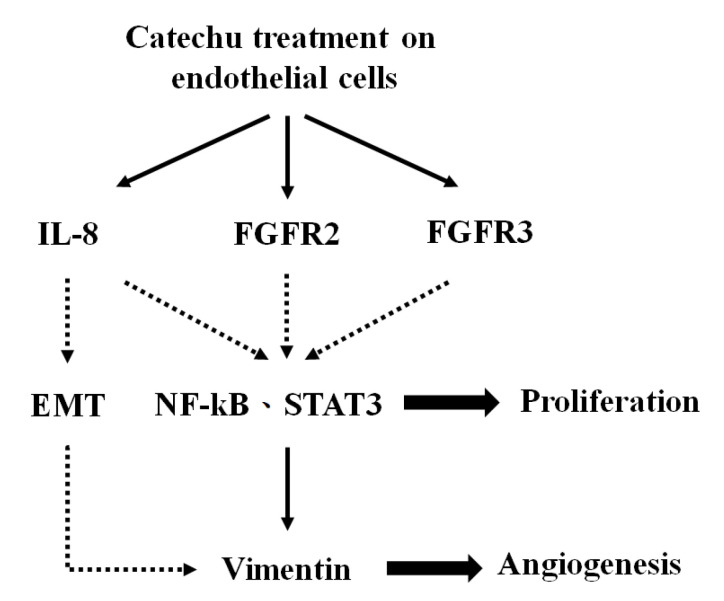
Summary of the possible mechanism underlying the angiogenic effect of catechu extracts in HMEC1 cells.

Vimentin, a well-known marker of EMT, is the most abundant intermediate filament protein in endothelial cells [27,28]. It engages in an array of cellular movements, and functions as an organizer of cell adhesion and migration through assembly and disassembly [29]. The involvement of vimentin in angiogenesis has been reported previously [30,31,32]. A summary of the possible mechanisms underlying the angiogenic effect of catechu extracts in HMEC1 is shown in Figure 5. Treatment with catechu induces the expression of FGFR2 and FGFR3 and, in turn, triggers downstream signaling of factors such as NF-kB and STAT3, which leads to the proliferation, adhesion, and migration of endothelial cells. Finally, various signaling molecules interact to induce vimentin expression and angiogenesis.

### 2.5. Cell Proliferation Assay

**Figure 6:**
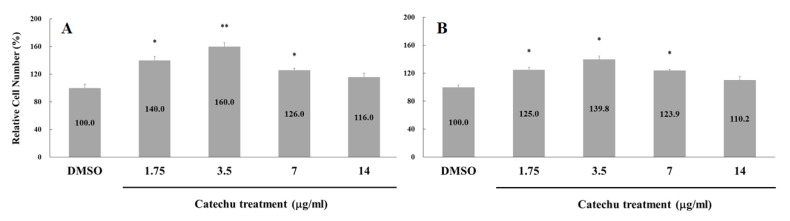
Effects of catechu extracts on (**A**) WS1 and (**B**) HaCaT proliferation. Increased cell proliferation was observed after treatment with catechu extracts for 24 h. Data are presented as means ± SD in three independent experiments. The *p*-value was calculated versus the control. Using the one-tailed test analysis, * indicates *p* < 0.05 and ** indicates *p* < 0.005 between different catechu concentrations.

The stimulatory effect of catechu extract on cell proliferation in both WS1 and HaCaT cells was measured. The increased percentages of cell proliferation by 1.75, 3.5, 7, and 14 μg/mL catechu extracts at 24 h for WS1 cells were 40.0 ± 5.7%, 60.0 ± 5.7%, 26.0 ± 2.8%, and 16.0 ± 5.7%, respectively (Figure 6A), and for HaCaT cells were 25.0 ± 3.2%, 39.8 ± 4.8%, 23.9 ± 1.6%, and 10.2 ± 4.8%, respectively (Figure 6B). For both cell types, the optimal treatment concentration of catechu was 3.5 μg/mL.

### 2.6. In Vitro Wound Healing Assay

**Figure 7:**
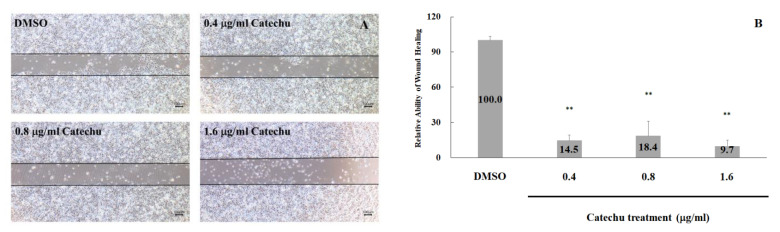
Effects of catechu extracts on wound healing. HaCaT cells were treated with 0.4, 0.8, and 1.6 mg/mL catechu extracts for 48 h. (**A**) The black lines denote the size of the cell-free gap at the beginning of the catechu treatment. HaCaT cells that migrated into the black lines were calculated using ImageJ after 48 h. The quantitation data of wound healing ability are shown in (**B**). Data are presented as means ± SD in three independent experiments. Statistical significance: ** *p* < 0.005 versus the control group (Con) using the one-tailed test analysis. The DMSO group was the solvent control.

During wound healing, keratinocytes migrate toward the margins of the wound. Therefore, the in vitro wound healing activity of crude catechu extracts was investigated. HaCaT cells were treated with 0.4, 0.8, and 1.6 μg/mL of catechu extracts for 48 h. As the incubation time increased, the number of cells that migrated into the gray gap area delineated by the black line also progressively increased (Figure 7A). The migration of HaCaT cells was inhibited by approximately 85.5%, 81.6%, and 90.3% in the presence of 0.4, 0.8, and 1.6 μg/mL of catechu extracts, respectively (Figure 7B).

## 3. Materials and Methods

### 3.1. Materials

HPLC-grade acetonitrile and methanol were obtained from ECHO Chemical Co. Ltd. (Miaoli, Taiwan). Pronase (catalog number: PRON-RO), 1-phenyl-2-thiourea (PTU), and molecular biology-grade DMSO were purchased from Sigma-Aldrich (St. Louis, MO, USA). Catechin and epicatechin were obtained from Tokyo Chemical Industry Co. Ltd. (Tokyo, Japan). Quercetin was purchased from Alfa Aesar (Ward Hill, MA, USA). Catechu (dried evaporated decoction from *Uncaria gambir* Roxb) was obtained from Hing Zong Co., Ltd. (Kaohsiung, Taiwan) (batch number: 213N-107-02). Raw materials were imported from Singapore (Figure 1).

### 3.2. HPLC Analysis of Catechu from Uncaria Gambir

A Hitachi L-7100 quaternary gradient pump and an L-7450 photodiode array detector were used to conduct HPLC analysis. Chromatographic data collection and processing, and pump and detector control were controlled using HSM software from Hitachi. A reverse-phase HPLC column (μBondapak^TM^ C18 Column, 125 Å, 10 μm, 3.9 × 300 mm from Waters (Milford, MA, USA) was used for analysis. The HPLC separation conditions for catechin, epicatechin, and quercetin were modified from previous studies [2,33]. Catechin and epicatechin were analyzed using a gradient elution of A (H_2_O containing 0.1% formic acid), B (10% CH_3_CN containing 0.1% formic acid), and C (90% CH_3_CN containing 0.1% formic acid) at a flow rate of 1 mL/min [1]. The elution program is shown in Table 3. The UV detection wavelength was 280 nm.

### 3.3. Preparation of Crude Catechu Extracts

The catechu blocks were then ground into a fine powder. One gram of catechu powder was dissolved in methanol (100 mL). After soaking overnight at room temperature, the methanol extracts were transferred to a new vial. Another 40 mL of methanol was added and the mixture was extracted at room temperature overnight. The final volume of the extract was adjusted to 250 mL using methanol. Undissolved particles were removed by centrifugation at 2500× *g* for 10 min at room temperature and filtered using a 0.22 μm PVDF syringe filter. The filtrate was dried in a 1.5 mL Eppendorf tube using Savant SpeedVac™ Vacuum Concentrators (Thermo Fisher Scientific Inc. Waltham, MA, USA). The solids were dissolved in DMSO for the subsequent in vivo and in vitro experiments.

### 3.4. In Vivo Zebrafish Embryo Angiogenesis Assay

The maintenance and treatment of zebrafish *Danio rerio* AB strains, wild-type, and *Tg* (*fli1-EGFP*) transgenic strains have been described previously [6,34]. Pronase (1 mg/mL) and PTU (1.5 mg/mL) were used to treat fish embryos at 12 and 24 h post-fertilization (hpf). At 55 hpf, fish embryos were moved to a 12-well plate, and five embryos were tested together in each well. Catechu extracts in DMSO were diluted in E3 buffer (5 mM NaCl, 0.17 mM KCl, 0.33 mM CaCl_2_, and 0.33 mM MgSO_4_, pH 7.2) and the final working solution contained 0.1% DMSO only for the experimental groups. Mortality and angiogenic phenotypes were observed at 72 hpf. Embryo blood vessel images were captured using a fluorescent stereomicroscope (Model: NSZ818; Nexcope, Ningbo, China) equipped with a CCD camera. Phenotypic quantification of sprout length was performed using WenXin WximageView software (accessed on 11 Oct 2021) (http://www.wenxin.net.tw/TW/home/Default.asp). The Institutional Animal Care and Use Committee of Tzu Chi Hospital approved all animal experiments in this study (case number: 111-15).

### 3.5. Cell Culture

Human keratinocytes (HaCaT) were cultured in Dulbecco’s modified Eagle’s medium (DMEM) containing 10% FBS. Human fibroblast WS1 cells were cultured in Eagle’s minimum essential medium containing 10% FBS. Human umbilical vein endothelial cells (HUVEC) were cultured in an endothelial cell growth medium-2 bulletkit (Lonza, Basel, Switzerland). Human dermal microvascular endothelial cells (HMEC-1) were cultured in MCDB131 medium containing 10% FBS, 10 ng/mL epidermal growth factor, 1 μg/mL hydrocortisone, and 10 mM L-glutamine. HaCaT and HMEC-1 cells were kindly provided by Professor Shinn-Jong Jiang (Department of Biochemistry, Tzu Chi University). WS1 and HUVEC were purchased from the Bioresource Collection and Research Center (Hsinchu, Taiwan). All the cell lines were incubated at 37 °C in a 5% CO_2_ atmosphere.

### 3.6. In Vitro Tube Formation Angiogenesis Assay

Matrigel (10 µL; BD Biosciences, Bedford, MA, USA) was applied to the inner well of a 15-well μ-slide (ibidi USA, Fitchburg, WI, USA). After gel polymerization, the μ-slides were placed inside a CO_2_ incubator for at least 30 min. Fifty microliters of 1 × 10^4^ HUVEC or HMEC-1 cells were mixed with the corresponding medium containing various concentrations of catechu extracts and incubated for 24 h [1,35]. At that time point, the cell tube or network formation was observed and photographed under a microscope.

### 3.7. Reverse Transcription and Quantitative Polymerase Chain Reaction

HMEC-1 cells were treated with 1 ng/mL of crude catechu extract. RNA was collected after treatment with catechu for 1, 2, 4, 6, and 24 h, and isolated using a Gene-spin Total RNA Purification Kit (Protech Technology Enterprise Co., Ltd., Taipei, Taiwan). Reverse transcription was performed using the MMLV Reverse Transcription Kit (Protech Technology Enterprise Co., Ltd., Taipei, Taiwan). All the experimental steps were carefully performed according to the manufacturer’s instructions [36]. The levels of β-actin mRNA were used to normalize the mRNA expression levels of the target genes. The primers used are listed in Table 4.

### 3.8. Cell Proliferation Assay

WS1 and HaCaT cells (4 × 10^4^/well) were seeded in 24-well plates and mixed with different concentrations of catechu extract for 24 h. Adherent cells were dissociated from the surface by treatment with 0.5% trypsin for 2–5 min. The number of cells was counted using a hemocytometer [36].

### 3.9. In Vitro Wound Healing Assay

Culture-inserts 2 well (Ibidi Gmbh, Gräfelfin, Germany) were placed on the center area of the well in 12-well cell culture plates. HaCaT cells were seeded in each well and incubated overnight. With 100% cell confluence, the culture inserts were removed, and the culture medium was changed to a serum-free medium containing 0.4, 0.8, and 1.6 mg/mL of catechu extracts. The culture insert was removed at 0 h. The area of the cell-free gap zone was photographed and defined by drawing the lines. After 48 h, the cell-free gap zone was photographed again and the cells that migrated into the cell-free gap zone acted as an indicator of wound healing [5]. The cell number was counted manually using the ImageJ software. The results for each group were compared.

## 4. Conclusions

Catechin and epicatechin are well known for their antioxidant, anti-inflammatory, and antimicrobial activities. Due to the high amounts of catechin and epicatechin in catechu (>21%), it is reasonable to assume that these constituents also play an essential role in treating chronic diabetic wounds. Interestingly, the in vivo and in vitro angiogenic activity of catechu was first reported in this study. These results can ideally account for the intense blood flow flux at porcine excisional wound sites [2]. Unexpectedly, catechu extracts inhibited the migration of HaCaT cells. However, the wound-healing activities of the other three herbal constituents in “Jinchuang ointment,” dragon blood, frankincense, and myrrh, have been reported previously [44,45,46]. In summary, these results provide insights into the therapeutic activity of catechu extracts in chronic diabetic wounds.

## Figures and Tables

**Table 3:** The gradient elution program for HPLC analysis of catechu extracts.

Time (min)	Eluent (B, C%)
0	0%, 0%
3.5	100%, 0%
18	96%, 4%
23	0%, 0%

**Table 4:** Primer sequences for RT-qPCR of target genes.

Gene	Primer Sequence	Reference
FGFR2	FORWARD: GAGAAGGAGATCACGGCTTC REVERSE: AAGTCTGGCTTCTTGGTCGT	[37]
FGFR3	FORWARD: GCCTCCTCGGAGTCCTTG REVERSE: GCCTCCTCGGAGTCCTTG	[38]
IL8	FORWARD: CTCTCTTGGCAGCCTTCCTGA REVERSE: CCCTCTGCACCCAGTTTTCCTT	[39]
NF-kB	FORWARD: CCTGGATGACTCTTGGGAAA REVERSE: TCAGCCAGCTGTTTCATGTC	[40]
STAT3	FORWARD: CATATGCGGCCAGCAAAGAA REVERSE: ATACCTGCTCTGAAGAAACT	[41]
Vimentin	FORWARD: TCAATGTTAAGATGGCCCTTG REVERSE: TGAGTGGGTATCAACCAGAGG	[42]
β-actin	FORWARD: AGAGCTACGAGCTGCCTGAC REVERSE: AGCACTGTGTTGGCGTACAG	[43]

## Data Availability

Data are contained within the article.

## References

[1] Ho T.J., Jiang S.J., Lin G.H., Li T.S., Yiin L.M., Yang J.S., Hsieh M.C., Wu C.C., Lin J.G., Chen H.P. (2016). The In Vitro and In Vivo Wound Healing Properties of the Chinese Herbal Medicine “Jinchuang Ointment”. Evid. Based Complement. Alternat. Med..

[2] Ho T.J., Chen J.K., Li T.S., Lin J.H., Hsu Y.H., Wu J.R., Tsai W.T., Chen H.P. (2020). The curative effects of the traditional Chinese herbal medicine “Jinchuang ointment” on excisional wounds. Chin. Med..

[3] Hsu W.H., Tsai W.T., Hung S.J., Cheng H.C., Hsu C.H., Ho T.J., Chen H.P. (2019). Treatment of leprosy wounds with “jinchuang ointment”, a traditional Chinese herbal medicine complex. Lepr. Rev..

[4] Wu C., Cai X.Q., Chang Y., Chen C.H., Ho T.J., Lai S.C., Chen H.P. (2019). Rapid identification of dragon blood samples from Daemonorops draco, Dracaena cinnabari and Dracaena cochinchinensis by MALDI-TOF mass spectrometry. Phytochem. Anal..

[5] Li F., Jiang T., Liu W., Hu Q., Yin H. (2016). The angiogenic effect of dracorhodin perchlorate on human umbilical vein endothelial cells and its potential mechanism of action. Mol. Med. Rep..

[6] Krishnaraj P., Chang Y., Ho T.J., Lu N.C., Lin M.D., Chen H.P. (2019). In vivo pro-angiogenic effects of dracorhodin perchlorate in zebrafish embryos: A novel bioactivity evaluation platform for commercial dragon blood samples. J. Food Drug Anal..

[7] Committee on Chinese Medicine and Pharmacy (2016). Taiwan Herbl Pharmacopeia English Version.

[8] State Pharmacopoeia Commission of the PRC (2015). Pharmacopoeia of the People’s Republic of China.

[9] Wu L.-C. (2006). Studies on the Chemical Specification of Forty Chinese Crude Drugs and Quantitative Analysis of Major Ingredients of Ercha and Qingdai. Master’s Thesis.

[10] State Pharmacopoeia Commission of the PRC (2015). Pharmacopoeia of the People’s Republic of China.

[11] Sazwi N.N., Nalina T., Abdul Rahim Z.H. (2013). Antioxidant and cytoprotective activities of Piper betle, Areca catechu, Uncaria gambir and betel quid with and without calcium hydroxide. BMC Complement. Altern. Med..

[12] Oswari L., Hidayat R., Fatmawati F., Hayati L., Alisa B.S. (2019). Gambir Extract (Uncaria Gambir) Decreases Inflammatory Response and Increases Gastric Mucosal Integrity in Wistar Rats—Model Gastritis. Open Access Maced. J. Med. Sci..

[13] Abral H., Kurniawan A., Rahmadiawan D., Handayani D., Sugiarti E., Muslimin A.N. (2022). Highly antimicrobial and strong cellulose-based biocomposite film prepared with bacterial cellulose powders, Uncaria gambir, and ultrasonication treatment. Int. J. Biol. Macromol..

[14] Teixeira A.M., Sousa C. (2021). A Review on the Biological Activity of Camellia Species. Molecules.

[15] Mendonça R.J., Coutinho-Netto J. (2009). Cellular aspects of wound healing. An. Bras. Dermatol..

[16] Koch A.E., Polverini P.J., Kunkel S.L., Harlow L.A., DiPietro L.A., Elner V.M., Elner S.G., Strieter R.M. (1992). Interleukin-8 as a macrophage-derived mediator of angiogenesis. Science.

[17] Schönbeck U., Brandt E., Petersen F., Flad H.D., Loppnow H. (1995). IL-8 specifically binds to endothelial but not to smooth muscle cells. J. Immunol..

[18] Strieter R.M., Polverini P.J., Kunkel S.L., Arenberg D.A., Burdick M.D., Kasper J., Dzuiba J., Van Damme J., Walz A., Marriott D. (1995). The functional role of the ELR motif in CXC chemokine-mediated angiogenesis. J. Biol. Chem..

[19] Matsushima K., Yang D., Oppenheim J.J. (2022). Interleukin-8: An evolving chemokine. Cytokine.

[20] Farjood F., Ahmadpour A., Ostvar S., Vargis E. (2020). Acute mechanical stress in primary porcine RPE cells induces angiogenic factor expression and in vitro angiogenesis. J. Biol. Eng..

[21] Cane G., Ginouvès A., Marchetti S., Buscà R., Pouysségur J., Berra E., Hofman P., Vouret-Craviari V. (2010). HIF-1alpha mediates the induction of IL-8 and VEGF expression on infection with Afa/Dr diffusely adhering E. coli and promotes EMT-like behaviour. Cell. Microbiol..

[22] Beck L., D’Amore P.A. (1997). Vascular development: Cellular and molecular regulation. FASEB J..

[23] Klein S., Bikfalvi A., Birkenmeier T.M., Giancotti F.G., Rifkin D.B. (1996). Integrin regulation by endogenous expression of 18-kDa fibroblast growth factor-2. J. Biol. Chem..

[24] Klein S., Giancotti F.G., Presta M., Albelda S.M., Buck C.A., Rifkin D.B. (1993). Basic fibroblast growth factor modulates integrin expression in microvascular endothelial cells. Mol. Biol. Cell.

[25] Seghezzi G., Patel S., Ren C.J., Gualandris A., Pintucci G., Robbins E.S., Shapiro R.L., Galloway A.C., Rifkin D.B., Mignatti P. (1998). Fibroblast growth factor-2 (FGF-2) induces vascular endothelial growth factor (VEGF) expression in the endothelial cells of forming capillaries: An autocrine mechanism contributing to angiogenesis. J. Cell Biol..

[26] Hart K.C., Robertson S.C., Kanemitsu M.Y., Meyer A.N., Tynan J.A., Donoghue D.J. (2000). Transformation and Stat activation by derivatives of FGFR1, FGFR3, and FGFR4. Oncogene.

[27] Evans R.M. (1998). Vimentin: The conundrum of the intermediate filament gene family. Bioessays.

[28] Osborn M., Geisler N., Shaw G., Sharp G., Weber K. (1982). Intermediate filaments. Cold Spring Harb. Symp. Quant. Biol..

[29] Ivaska J., Pallari H.M., Nevo J., Eriksson J.E. (2007). Novel functions of vimentin in cell adhesion, migration, and signaling. Exp. Cell Res..

[30] Eckes B., Colucci-Guyon E., Smola H., Nodder S., Babinet C., Krieg T., Martin P. (2000). Impaired wound healing in embryonic and adult mice lacking vimentin. J. Cell Sci..

[31] Eckes B., Dogic D., Colucci-Guyon E., Wang N., Maniotis A., Ingber D., Merckling A., Langa F., Aumailley M., Delouvée A. (1998). Impaired mechanical stability, migration and contractile capacity in vimentin-deficient fibroblasts. J. Cell Sci..

[32] Lundkvist A., Reichenbach A., Betsholtz C., Carmeliet P., Wolburg H., Pekny M. (2004). Under stress, the absence of intermediate filaments from Müller cells in the retina has structural and functional consequences. J. Cell Sci..

[33] Kamal S., Susanti M., Febriyenti, Zaini E., Hamidi D. (2022). Simultaneous TLC-densitometric analysis of catechin, pyrocatechol and quercetine in gambir block from Pesisir Selatan. Heliyon.

[34] Lawson N.D., Weinstein B.M. (2002). In vivo imaging of embryonic vascular development using transgenic zebrafish. Dev. Biol..

[35] Chiang J.H., Yang J.S., Lu C.C., Hour M.J., Chang S.J., Lee T.H., Chung J.G. (2013). Newly synthesized quinazolinone HMJ-38 suppresses angiogenetic responses and triggers human umbilical vein endothelial cell apoptosis through p53-modulated Fas/death receptor signaling. Toxicol. Appl. Pharmacol..

[36] Wu J.-R., Lu Y.-C., Hung S.-J., Lin J.-H., Chang K.-C., Chen J.-K., Tsai W.-T., Ho T.-J., Chen H.-P. (2021). Antimicrobial and Immunomodulatory Activity of Herb Extracts Used in Burn Wound Healing: “San Huang Powder”. Evid.-Based Complement. Altern. Med..

[37] Chen X., Song D. (2021). LncRNA MEG3 Participates in Caerulein-Induced Inflammatory Injury in Human Pancreatic Cells via Regulating miR-195-5p/FGFR2 Axis and Inactivating NF-κB Pathway. Inflammation.

[38] Blick C., Ramachandran A., Wigfield S., McCormick R., Jubb A., Buffa F.M., Turley H., Knowles M.A., Cranston D., Catto J. (2013). Hypoxia regulates FGFR3 expression via HIF-1α and miR-100 and contributes to cell survival in non-muscle invasive bladder cancer. Br. J. Cancer.

[39] Pina-Canseco Mdel S., Páez-Arenas A., Massó F., Pérez-Campos E., Martínez-Cruz R., Hernández-Cruz P., Majluf-Cruz A., Martínez-Cruz M., Pérez-Campos Mayoral L., Pérez-Santiago A.D. (2012). Protein C activation peptide inhibits the expression of ICAM-1, VCAM-1, and interleukin-8 induced by TNF-α in human dermal microvascular endothelial cells. Folia Histochem. Cytobiol..

[40] Yuan W., Sun Q., Jiang Y., Zhang X., Chen L., Xie C., Qin F., Chen Y., Lv H., Chen W. (2016). MiR-146a affects the alteration in myeloid differentiation induced by hydroquinone in human CD34(+) hematopoietic progenitor cells and HL-60 cells. Toxicol. Res. (Camb.).

[41] Goel S., Sahu S., Minz R.W., Singh S., Suri D., Oh Y.M., Rawat A., Sehgal S., Saikia B. (2018). STAT3-Mediated Transcriptional Regulation of Osteopontin in STAT3 Loss-of-Function Related Hyper IgE Syndrome. Front. Immunol..

[42] Li J., Dong L., Wei D., Wang X., Zhang S., Li H. (2014). Fatty acid synthase mediates the epithelial-mesenchymal transition of breast cancer cells. Int. J. Biol. Sci..

[43] Chen F., Zhang G., Yu L., Feng Y., Li X., Zhang Z., Wang Y., Sun D., Pradhan S. (2016). High-efficiency generation of induced pluripotent mesenchymal stem cells from human dermal fibroblasts using recombinant proteins. Stem Cell Res. Ther..

[44] Lu C.C., Yang J.S., Chiu Y.J., Tsai F.J., Hsu Y.M., Yin M.C., Juan Y.N., Ho T.J., Chen H.P. (2021). Dracorhodin perchlorate enhances wound healing via β-catenin, ERK/p38, and AKT signaling in human HaCaT keratinocytes. Exp. Ther. Med..

[45] Gebrehiwot M., Asres K., Bisrat D., Mazumder A., Lindemann P., Bucar F. (2015). Evaluation of the wound healing property of Commiphora guidottii Chiov. ex. Guid. BMC Complement. Altern. Med..

[46] Negahdari S., Galehdari H., Kesmati M., Rezaie A., Shariati G. (2017). Wound Healing Activity of Extracts and Formulations of Aloe vera, Henna, Adiantum capillus-veneris, and Myrrh on Mouse Dermal Fibroblast Cells. Int. J. Prev. Med..

